# Improving Psychiatric Hospital Care for Pediatric Patients with Autism Spectrum Disorders and Intellectual Disabilities

**DOI:** 10.1155/2012/685053

**Published:** 2012-06-19

**Authors:** Robin L. Gabriels, John A. Agnew, Carol Beresford, Mary Ann Morrow, Gary Mesibov, Marianne Wamboldt

**Affiliations:** ^1^School of Medicine University of Colorado, 13001 E 17th Place, Aurora, CO 80045, USA; ^2^Children's Hospital Colorado, 13123 E. 16th Avenue, Aurora, CO 80045, USA; ^3^University of North Carolina, Chapel Hill, 510 Meadowmont Circle, Suite 300, Chapel Hill, NC 27517, USA

## Abstract

Pediatric patients with autism spectrum disorders (ASD) and/or intellectual disabilities (ID) are at greater risk for psychiatric hospitalization compared to children with other disorders. However, general psychiatric hospital environments are not adapted for the unique learning styles, needs, and abilities of this population, and there are few specialized hospital-based psychiatric care programs in the United States. This paper compares patient outcomes from a specialized psychiatric hospital program developed for pediatric patients with an ASD and/or ID to prior outcomes of this patient population in a general psychiatric program at a children's hospital. Record review data indicate improved outcomes for patients in the specialized program of reduced recidivism rates (12% versus 33%) and decreased average lengths of inpatient stay (as short as 26 days versus 45 days). Available data from a subset of patients (*n* = 43) in the specialized program showed a decrease in irritability and hyperactivity behaviors from admission to discharge and that 35 previously undetected ASD diagnoses were made. Results from this preliminary study support specialized psychiatric care practices with this population to positively impact their health care outcomes.

## 1. Introduction

There is a growing need to establish and evaluate innovative specialized psychiatric healthcare programs for psychiatric patients diagnosed with an autism spectrum disorder (ASD) and/or an intellectual disability (ID). This population is at risk for psychiatric hospitalization due to the fact that psychiatric disorders are highly prevalent in individuals with an ID [1–3]. In addition, psychiatric comorbidities have been reported to be as high as 72% in a pediatric population of individuals with an ASD [4] with increased rates of psychopathology in children and adolescents who are diagnosed with both an ASD and an ID [5–7]. Specifically, there are high rates of affective (depression) and anxiety disorders in the ASD population [4, 7–10]. Factors that may put children with an ASD at higher risk for psychiatric hospitalization include living in a single-parent home, being diagnosed at an older age, engaging in self-injurious and aggressive behaviors, and being diagnosed with depression or obsessive compulsive disorder [11]. This information, combined with the rising ASD prevalence rates [12–14], suggests a growing need for psychiatric preventative and crisis care management options for the ASD population at all ages. An added complication is the fact that the ASD/ID population often lacks the social communication and cognitive capacity necessary to report internal physical or emotional experiences. These are capabilities often expected by psychiatric or medical personnel to complete successful psychiatric assessments or medical examinations.

General psychiatric hospital environments are not adapted for the unique learning styles, needs, and abilities of the pediatric ASD/ID population. (See [15] for a literature review of ASD learning styles and targeted behavioral intervention strategies). Unfamiliar settings (e.g., hospitals) or procedures can cause anxiety in these children, which may be expressed by an increase in disruptive or aggressive behaviors [16]. Impairments in social communication skills and intellectual abilities can contribute to the child's inability to understand expectations. Abnormal sensory responses to stimuli such as light, sounds, touch, and smell can make experiences in hospital settings very uncomfortable or even intolerable for many children, causing them to display tantrums or other symptoms of distress (e.g., self-injury or aggression). This population is also unlikely to respond positively to verbal intervention strategies (e.g., verbal reassurance, coaxing or explanations) that are typically used by psychiatric personnel. Concurrently, general psychiatric hospital personnel are not routinely trained to understand and respond effectively to the unique learning styles of this ASD/ID population [17] or to consider the impact that factors such as health (e.g., experience of pain) or environmental stimuli can have on an agitated psychiatric patient with special needs. Untrained psychiatric personnel present a high risk for inaccurate assessment of presenting crisis behaviors as well as inappropriate or excessive use of interventions such as seclusion/restraints, PRN medications, and high patient-to-staff care ratios. This lack of understanding not only puts this population at risk for limited, inappropriate, or ineffective care but also puts hospital staff at risk for harm.

Despite the high need for specialized hospital-based psychiatric care services for the ASD/ID population, these services are currently limited in the United States. A recent survey-based study found only nine ASD/ID specialty inpatient psychiatric programs, all geographically limited to the northeast [18]. Results from that study suggest that there has been an increase in the number of these specialized inpatient programs developed within the past 10 years. Based on a 12-month survey period, the average length of stay for these programs was 42.3 days (30.75 days with the removal of a 135 day outlier); they served patients' ages 4–21 years of age (mean age 12.72) with 62.5–87.5% having an ASD; the most common chief complaints were aggression and self-injury, and patient-to-staff ratios were high (i.e., three staff to four patients). The most common challenge identified by these programs was a lack of available community follow-up care services. The authors proposed that this may in part explain why many of these programs also have a continuum of care component such as a partial hospitalization or some other type of intensive outpatient service. Finally, all of the programs from this survey utilized some form of behavioral therapy combined with psychopharmacology [18].

The main objective of this retrospective chart review study was to determine if the introduction of a specialized psychiatric intervention program into a children's hospital setting improved psychiatric care outcomes (i.e., hospital lengths of stay and readmission rates) of patients diagnosed with an ASD and/or ID. Our secondary objective was to examine the population characteristics and behavioral (i.e., irritability and hyperactivity) changes from admission to discharge for patients treated in this newly developed specialized intervention program.

## 2. Methods

### 2.1. Population and Intervention Programs

The Children's Hospital Colorado (previously, The Children's Hospital) is associated with the University of Colorado School of Medicine. The specialty program evaluated in this study is the neuropsychiatric special care (NSC) program of the Children's Hospital Colorado within the Department of Psychiatry and Behavioral Sciences. This specialty psychiatric program was initiated in April 2004 to provide short-term inpatient and intensive day treatment (partial hospitalization) assessment and stabilization for children and adolescents diagnosed with an ASD and/or an ID who present with comorbid psychiatric and/or medical conditions. The intensive day treatment (partial hospitalization) component of the program was developed as an effort to decrease inpatient lengths of stay and repeated admissions. This provided an additional intensive care resource for patients and caregivers to practice skills in the home environment that had been learned in the inpatient setting. This intensive day treatment program is similar to some of the continuum of care components described by Siegel et al. [18]. Prior to the initiation of this specialty psychiatric program, the ASD/ID population was treated in the General Child and Adolescent Inpatient Psychiatric Unit at the hospital, and there was no partial hospitalization option. Given the social communication limitations of this population, the care on this general psychiatric unit primarily consisted of medication management, 1 : 1 staffing ratios, and limited involvement of the patient in general milieu activities or caregiver teaching activities.

Typically, patients diagnosed with an ASD and/or ID present to the Children's Hospital Colorado Psychiatric Emergency Department with behaviors that include an increase in mood instability (including suicidal or homicidal threats or actions), impulsivity, aggression towards others, destruction of property, or severe self-injury. Teasing out the underlying etiologies of these presenting symptoms from the patients' developmental disability can be difficult because **s**uch behaviors may not be part of the DSM IV-TR [19] diagnostic criteria of the Pervasive Developmental Disorders or Mental Retardation (MR) but may instead represent a *change *in patients' baseline level of developmental functioning.

The intervention approach of the NSC program was created to improve the process of assessing and targeting the underlying etiologies of presenting crisis behaviors in the ASD/ID population. This approach involves providing a structured environment with predictable routines and visual cues designed to help the patient better understand the environment and its expectations and also to avoid agitating patients unnecessarily by decreasing their need for physical prompting by unfamiliar hospital personnel. Multidisciplinary program staff is trained to (1) understand the distinctive ways individuals with an ASD/ID think and learn (i.e., the “culture of autism” [20]), (2) assess the issues underlying patients' presenting “tip of the iceberg” crisis behaviors, and (3) implement positive/proactive behavior management strategies [21, 22]. This approach aims to decrease the diagnostic “noise” of the developmental disability (e.g., communication frustration or limited adaptive living or cognitive skills) so that the psychiatric, medical, behavioral, and family issues contributing to or driving the current crisis for these patients can be determined and managed in an efficient manner. An additional aim of the NSC program is to provide structured activities similar to a community setting within the confines of the hospital setting in order to assess patients' levels of readiness for discharge and identify adaptations needed to increase patients' success in community settings.

The structured environment of the NSC program reflects the TEACCH (treatment and education of autistic and related communication handicapped children) model developed in the 1960s and early 1970s at the University of North Carolina, Chapel Hill, USA [23]. The TEACCH model is based upon cognitive-behavioral and social learning principles and aims to design environments that meet the unique learning style needs and preferences of the ASD population [23]. These structured environments are put in place to increase an individual's independent functioning and understanding of expectations in an effort to decrease undesirable, dangerous behaviors. There is empirical evidence that the introduction of environmental structure can help individuals with an ASD increase their attending behaviors and reduce challenging behaviors. (See [24, 25] for a review.)

Specifically, the structured environment of the NSC program involves organizing the environment to clearly define intervention areas for activities such as social group, independent leisure, relaxation/quiet, independent work, and work with staff times. The environmental organization is necessary to decrease the need for excessive physical prompting of the patient as the patient quickly begins to associate activities with specific locations. The NSC environment provides concrete visual cues (specific to patients' needs and ability levels) that include object, pictures, or written word cues for rules and instructions. Visual cues and instruction techniques include the use of daily and mini routine picture schedules, Social Stories [26], role play, error correction techniques paired with demonstration (e.g., staff say “Try again” and demonstrate the appropriate behavior response for patients), social praise for appropriate behaviors to increase patients' prosocial motivation, and visual road maps to help patients understand their choices and the outcomes of their behaviors. The structured environment of the NSC includes a consistent daily schedule and familiar routines to promote patients' experience of predictability. The daily schedule alternates less preferred patient activities (e.g., social groups) with patient-preferred activities (e.g., choice times) in order to promote patient motivation to participate in less preferred activities.

When this study was carried out, the NSC program had up to eight patients in the day treatment program and up to three inpatients at any one time. The staff-to-patient ratio in the day treatment service was one staff to two patients, and the staff-to-patient ratio on the inpatient service was typically 1 : 1. The program staff included a multidisciplinary treatment team led by a psychologist/program director (first author) and psychiatrist/medical director (third author). The psychologist's role was to develop cognitive-behavioral interventions, and the psychiatrist's role was to collaborate on medical and psychiatric concerns. Each family worked with a team of NSC program staff, which included a psychologist, psychiatrist, mental health counselor, registered nurse, and licensed mental health therapist, who functioned as the case manager/family support therapist. A pediatrician provided weekly consultation to the team, and other disciplines within the general hospital setting (including neurology, gastroenterology, genetics, endocrinology, nutrition, physical therapy, and dentistry) were routinely consulted. An occupational therapist and speech therapist were on site to address specific sensory-motor and communication needs of patients on a case-by-case basis. Creative arts' therapists (music, art, movement, and yoga) provided weekly group activities. Patients received a full range of routine activities that included daily living (chore and daily living routines), independent of work and leisure routines, social groups, and work with staff academic routines.

The intervention process of the NSC program began with an admission process that involved a review of caregiver reports of a patient's medical, psychiatric, and academic assessment history along with the patient's strengths, needs, and behavioral symptoms using the Child and Caregiver Information Form (CCIF) [27] as well as the Aberrant Behavior Checklist-Community (ABC-C) [28]. The admission process also involved a family art therapy evaluation of family issues and interactive patterns [29]. Based on this information, the NSC program generated individual patient goals and positive behavioral management interventions. In general, patient goals targeted behaviors in the following areas: increasing self-regulation skills, decreasing aggression and self-harm, increasing attending skills, and increasing positive social interactions with peers and family. For example, patients were taught self-regulation skills using strategies that included how to rate gradual changes in their emotions using a 1 (calm) to 4 (extremely upset) rating scale and when to ask to take time away in a designated quiet/calming area based on slight increases in their level of agitation. Other self-regulation teaching strategies involved providing patients with calming fidget toys during less preferred activity times (e.g., waiting times or social groups), reviewing a visual roadmap with patients of their behavior choices and outcomes, and engaging patients in creating Social Stories [26] to outline ways to cope with events. Throughout patients' treatment, families and community providers spent time in the milieu setting learning how to implement intervention plans specific to patients' developmental and emotional strengths and needs. Caregivers completed another ABC-C form regarding the child's behavior status at discharge.

### 2.2. Chart Review

This retrospective study was approved by the Colorado Multiple Institution Review Board (COMIRB), and patient/guardian consent was waived due to the fact that patient data was deidentified prior to collecting data for this study. The psychiatric hospital medical records of pediatric patients diagnosed with anASD and/or ID were reviewed from two different time and intervention periods: a general psychiatric pediatric inpatient program from 10/2001–11/2002 and for a one-year period that occurred five years following the inception of the NSC psychiatric program (all of 2009). For the chart review of October 1, 2001 to November 30, 2002 time period, a total of 20 patients were initially identified because they were on a list of patients kept by nursing staff who received a “special” behavior plan during their psychiatric admission. This “special” behavior plan was routinely implemented for patients who could not tolerate the general psychiatric milieu program. These patients included those with ASD and/or ID but could have also included patients with acute psychosis, encephalitis, or other acute medical concerns. Hospital administration (including the senior author) initially requested a clinical survey of these patient charts (by the first author) in 2003 in an effort to identify the need to establish a specialized psychiatric unit for this ASD/ID population who were placing extraordinary demands on hospital personnel and financial resources.

For both time periods, the abstracted clinical data included patients' gender, age at program admission, diagnoses (developmental, psychiatric, and medical), length of hospital stay, and readmission dates during the specified time period reviewed. Additional data abstracted from the NSC program patient records during the 2009 time period included the ABC-C [28], a 58-item symptom checklist for assessing problem behaviors (irritability, lethargy, stereotypic behavior, hyperactivity, and inappropriate speech) of children and adults with developmental disabilities. The ABC-C data is routinely collected for all patients in this program. During the time period of this study, only a subset (*n* = 43) of the ABC-C forms as completed by a consistent caregiver for patients at admission and discharge from the NSC day treatment program.

## 3. Statistical Considerations

Patients' demographic data (gender, age at program admission, diagnoses, length of hospital stay, and readmission information) was summarized using means and standard deviations. The admission and discharge caregiver report of patients' aberrant behavior symptoms on the ABC-C irritability and hyperactivity subscales as assessed by comparing the two data points (admission and discharge) using paired *t*-tests.

## 4. Results

Data from a total of 12 patient charts from the general psychiatric inpatient program (October 1, 2001–November 30, 2002) were included in this study because these patients had a documented ASD and/or ID diagnosis from a hospital psychiatrist. Chart data from all patients who were admitted to the NSC inpatient and day treatment (*n* = 105) were reviewed for 2009 (January 1, 2009–December 31, 2009). Of the 105 patients who were in the NSC program, 79 only attended the day treatment program, and the remaining 26 attended the inpatient program and then stepped down to attend the day treatment program. Of these 105 patients, only 11 patients did not have a diagnosis of ASD or an ID. These patients were referred to the NSC program for concerns that they may have ASD or a cognitive issue that interfered with their ability to participate in the regular psychiatric inpatient and day treatment programs. Patient demographics and clinical characteristics are described in detail in Table 1. Briefly, patients from both time periods varied in age from 3 to 18 years of age and presented with a range of comorbid medical (e.g., GERD, hypothyroid from lithium medication, asthma, chronic constipation, seizures, precocious puberty, pica, eczema, migraines, obesity, type 1 diabetes, severe dystonia, and sleep apnea) and psychiatric diagnoses (e.g., mood disorders, anxiety disorders, and psychotic disorders).

The outcome variables for this study included the mean length of stay and recidivism rates to the Children's Hospital Colorado within the timeframe considered by the study. For the purposes of this study, recidivism is defined as a return to the same program during the study period, so October 1, 2001–November 30, 2002 for the general psychiatric pediatric inpatient program or calendar year 2009 for the NSC program. As is shown in Table 1, the average length of stay for inpatients decreased from 45 inpatient days in the pre-NSC program to 26 days in the NSC inpatient program. The length of stay for NSC patients attending only the day treatment program was 12.6 days. The recidivism rate for inpatients decreased from 33.3% in the pre-NSC program to 11.5% in the NSC inpatient program. The recidivism rate for patients in only the NSC day treatment program was 10.3%.

For almost half of the patients in the NSC portion of the study (*n* = 43), the ABC-C was completed at admission and discharge by a consistent caregiver. Data from the ABC-C irritability and hyperactivity subscales were analyzed to characterize any changes in these aberrant behaviors. These data demonstrated an average of 8.3 point decline on the irritability subscale (from 23.1 to 14.8; *P* < .001) and a 7.9 point decline on the hyperactivity subscale (from 26.1 to 18.2; *P* = .003). The age range of this subset of this NSC patient population was 5–17 years, and the average age was 11.2 years. For comparison, in a community-based sample of children diagnosed with an ASD (ages 6–16 years) who were enrolled in a treatment outcome study of the effects of therapeutic horseback riding, ABC-C irritability scores declined by 7.3 points and hyperactivity scores declined by 6.6 points before to after intervention. Both of these were found to be significant at *P* < .001 in that study [30].

Patients who presented to the NSC program with chronic social and communication issues and who met the autism spectrum screening cutoffs on the Social Communication Questionnaire (SCQ) [31] were referred by their treatment team to rule out an autism spectrum diagnosis. The diagnostic evaluation included using the Autism Diagnostic Observation Scale [32, 33] and the Autism Diagnostic Interview (ADI-R) [34] during the NSC program to clarify diagnostic implications of patients' presenting behavioral issues. During the course of the NSC portion of the study, 35 patients out of the total patient population of 105 received a previously undetected novel diagnosis of an ASD. These patients ranged in age from 6 to 16 years with an average age of 10.3 years.

## 5. Discussion 

Although this is a preliminary study, results suggest that the introduction of a specialized psychiatric program designed to meet the unique needs of the ASD/ID pediatric population resulted in a clinically significant decline of inpatient lengths of stay and recidivism rates along with a substantial increase in the number of ASD/ID patients served. These results indicate that, compared to this hospital's previous practices of serving this special population on a general psychiatric unit, the NSC program was more successful in quickly assessing and stabilizing patients so they could leave the hospital and return to their communities. In addition, the comparison of the NSC program admission to discharge caregiver reports of patients' symptoms of irritability and hyperactivity showed a notable decline of approximately eight points on each ABC-C subscale after treatment in the NSC program. Further, many previously undetected ASD diagnoses were made in the NSC program during 2009, suggesting that the NSC may be providing an additional avenue for obtaining more accurate diagnoses leading to targeted treatment recommendations for their community settings. This may reduce the need for repeating psychiatric hospitalizations in the future.

The results from this study are limited by relying on data from case notes and the fact that this study only examined patients' recidivism rates during a particular window of time. Specifically, there is a potential inflation bias of results from the first time period (general psychiatric pediatric inpatient program from 10/2001–11/2002) due to the fact that the only ASD/ID patients included in this study were those who were quite challenging, and these data do not account for the possibility that there may have been higher functioning and less challenging ASD patients on the unit. An additional limitation is the fact that, for the second time period (NSC program in 2009), ABC-C data from a consistent caregiver at patients' admission and discharge were not available for all patients admitted to the NSC program.

Although the present study does not attempt to make recommendations about the generalizability of these treatment methods and outcomes to other settings, this preliminary study does lend support for the development of specialized psychiatric hospital programs for individuals diagnosed with an ASD and/or ID to improve their psychiatric healthcare outcomes.

The unique needs of the ASD/ID population require specialized psychiatric programs that involve assessment of the “root causes” of the patient's symptoms along with engaging families in the intervention and discharge planning process [11, page 1064]. The high prevalence of ASD patients in the NSC inpatient program (76.9%), similar to that reported by other specialty inpatient programs (62.5–87.5%) [18], indicates there is a demand for such services.

## Figures and Tables

**Table 1:** Demographics and outcome measures.

Category	10/2001–11/2002 Psychiatric inpatient program	1/2009–12/2009 NSC inpatient program	1/2009–12/2009 NSC day program	NSC patients who completed admission and discharge ABC-C data
Number or patients	12 (10 males)	26 (21 males)	79 (62 males)	43 (33 males)
Mean age (range)	13.7 (3–19)	12.4 (5–17)	10.7 (5–17)	11.2 (5–17)
# Diagnosed ASD (with and without an ID)	6 (50%)	20 (76.9%)	60 (75.9%)	34 (79.1%)
# Diagnosed ID without an ASD	6 (50%)	4 (15.4%)	8 (10.1%)	6 (14.0%)
Mean # comorbid medical diagnoses per patient	0.75	1.46	1.17	1.33
Mean # comorbid psychiatric diagnoses per patient	2.67	1.58	1.55	1.67
Mean # days of stay (range)	45 (13–180)	26.1 (10–56)	12.6 (1–41)	13.2 (1–41)
Recidivism rate^∗^	33.3%	11.5%	10.3%	20.9%

*Recidivism-patients readmitted to the NSC program within the 2009 calendar year.
